# Predator Presence and Vegetation Density Affect Capture Rates and Detectability of *Litoria aurea* Tadpoles: Wide-Ranging Implications for a Common Survey Technique

**DOI:** 10.1371/journal.pone.0143733

**Published:** 2015-11-25

**Authors:** Madeleine R. Sanders, Simon Clulow, Deborah S. Bower, John Clulow, Michael J. Mahony

**Affiliations:** School of Environmental and Life Sciences, University of Newcastle, Newcastle, NSW, Australia; Ben-Gurion University of the Negev, ISRAEL

## Abstract

Trapping is a common sampling technique used to estimate fundamental population metrics of animal species such as abundance, survival and distribution. However, capture success for any trapping method can be heavily influenced by individuals’ behavioural plasticity, which in turn affects the accuracy of any population estimates derived from the data. Funnel trapping is one of the most common methods for sampling aquatic vertebrates, although, apart from fish studies, almost nothing is known about the effects of behavioural plasticity on trapping success. We used a full factorial experiment to investigate the effects that two common environmental parameters (predator presence and vegetation density) have on the trapping success of tadpoles. We estimated that the odds of tadpoles being captured in traps was 4.3 times higher when predators were absent compared to present and 2.1 times higher when vegetation density was high compared to low, using odds ratios based on fitted model means. The odds of tadpoles being detected in traps were also 2.9 times higher in predator-free environments. These results indicate that common environmental factors can trigger behavioural plasticity in tadpoles that biases trapping success. We issue a warning to researchers and surveyors that trapping biases may be commonplace when conducting surveys such as these, and urge caution in interpreting data without consideration of important environmental factors present in the study system. Left unconsidered, trapping biases in capture success have the potential to lead to incorrect interpretations of data sets, and misdirection of limited resources for managing species.

## Introduction

Estimates of abundance and distribution for populations and species are fundamental metrics for understanding and managing wildlife [1]. However, any technique-related bias in the sampling of individuals has the potential to affect the accuracy of population estimates. Identifying factors that affect the success of a sampling technique is important to minimise bias that results from non-random and non-independent sampling in order to make accurate inferences about population dynamics. Minimising bias is particularly important for population monitoring, which aims to detect trends in populations [2]. Knowledge of any bias associated with sampling techniques is crucial for confidently drawing conclusions about population trends.

Passive trapping is a common sampling technique for capturing animals, particularly for species that are cryptic or difficult to survey by other means [3–5]. However, capture success can be influenced by behaviour, which in turn affects detectability and capture rate. Such changes in behaviour are particularly problematic for trapping techniques if the altered behaviour influences capture success. For example, salamander surface activity (and hence capture rate and detectability) varies depending on landscape variables such as topography, season, humidity and climate [5]. Thus, capture rates and detection probability can change within a population due to behavioural shifts over small spatial or temporal scales despite the use of a standardised trapping technique. Despite the potential for biased estimates, passive trapping devices continue to be widely used because they are simple and inexpensive, and can be replicated easily [6, 7].

Funnel trapping is a popular method that has been used for many years to assess the abundance and spatial distribution of aquatic vertebrates, particularly fish [3, 8, 9] and amphibian larvae. When funnel trapping in the natural environment, it is important to identify whether environmental factors influence the capture rate to account for bias in subsequent population estimates based on the trapping data. Studies that use count indices (e.g., mark-recapture) assume that individuals have the same capture probabilities for the duration of the sampling event, however, the assumption of equal catchability rarely holds true [10–12]. In addition, trapping techniques are rarely tested for sources of variability that are due to trapping biases [13]. Traps may be the only technique available in some cases, so understanding factors that influence their effectiveness is crucial.

Environmental factors such as predator presence and vegetation density can influence fish behaviour [14–16] and recent studies have linked these changes in fish behaviour to capture success in traps [17, 18]. Similarly, these environmental factors also change tadpole behaviour [19–26], but little is known about how the change in behaviour affects subsequent capture success. Our objective was to investigate the effects that behavioural plasticity, induced by common environmental parameters, might have on the capture success of tadpoles using a common trapping technique (funnel-trapping). Specifically, we aimed to determine the influence of a predatory fish and the influence of high and low vegetation density on the capture rate and detectability of tadpoles. We hypothesised that these common environmental factors would trigger behavioural plasticity, which in turn would create heterogeneous capture rates and affect detection probabilities and population estimates.

## Materials and Methods

### Collection and husbandry of study species

Our study design incorporated a tadpole (endangered green and golden bell frog, *Litoria aurea*) and a predatory fish (plague minnow, *Gambusia holbrooki*). These species often occur sympatrically in freshwater wetlands in south-eastern Australia, and *G*. *holbrooki* predates heavily on the eggs and tadpoles of *L*. *aurea* [27, 28]. Ethics approval was granted by the University of Newcastle Animal Care and Ethics Committee (A-2008-165) and NSW National Parks scientific licence (SL100190).

We used tadpoles from captive adult *L*. *aurea* breeding stock held at the University of Newcastle in December, 2012 (3 spawns in total). We reared tadpoles in cylindrical polyethylene tubs (45 cm height, 60 cm diameter), which were located in an outdoor animal holding facility. Tadpoles were exposed to natural ambient temperatures and day-night cycle. We fed tadpoles boiled lettuce and trout pellets (Ridley Aqua-feed, Ridley AgriProducts Pty Ltd, Narangba, Queensland, Australia) *ad libitum*, and maintained them until they reached at least Gosner developmental stage 25 [29]. Gosner stage at the time of the experiment ranged from 26 to 36, and the snout-vent length (SVL) from 10 mm to 20 mm (n = 480). An even mix of tadpoles from each spawn was used for each run of the experiment, with a range of sizes to incorporate natural variability.

We used a dip net to collect fish from a pond on the grounds of the University of Newcastle one hour prior to commencing the experiment, and immediately transferred them into the experimental mesocosms. The individuals captured consisted of a mix of males and females and were assigned haphazardly to experimental treatments.

### Experimental design and technique

**Fig 1:**
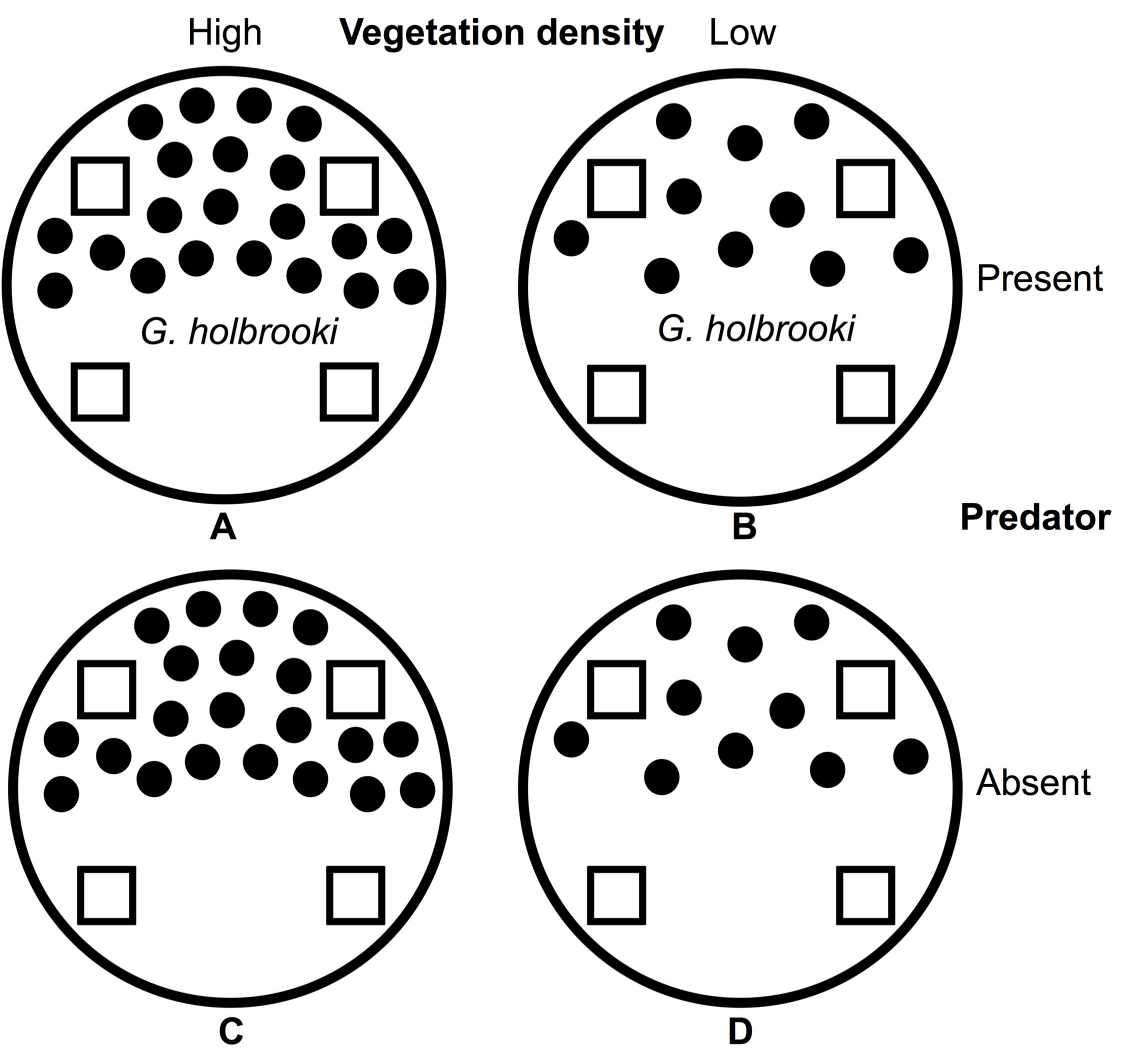
Experimental design of mesocosms. 2 x 2 full factorial design with vegetation (high or low density) and predator (*Gambusia holbrooki* present or absent) as factors to make up four treatments (A, B, C, D). Traps are represented by squares (n = 4 per mesocosm).

Our experimental design was a 2 x 2 full factorial design with vegetation density and predator presence as factors (Fig 1). We conducted experiments in 4 cylindrical polyethylene mesocosms (3.5 m diameter, 1 m height, 10,000 L volume; Duraplus aquapoly aquaculture tubs, Newcastle) that were filled to 0.8 m with rainwater and assigned either high or low density ‘vegetation’ patches. We created the different ‘vegetation’ patches with small (<10 mm diameter) bamboo stakes at high and low density. We constructed patches from 60 cm x 47 cm x 2 cm wooden boards with 60 cm high bamboo stakes and used non-toxic epoxy glue to stick stakes into drilled holes. High density vegetation contained 150 stakes per board, while low density contained 20 stakes per board. We fashioned the bamboo stakes to imitate the stems of emergent vegetation (such as *Baumea articulata* and *Schoenoplectus validus)* that are common in ponds that both species inhabit. In total, we placed 3 boards containing stakes side-by-side at one end of the mesocosm; each board was weighted down with 3 bricks. We placed 100 predatory fish into one of each of the low and high density vegetation mesocosms to create the ‘predator present’ treatments, with the remaining two mesocosms staying fish free. We did not remove fish from mesocosms after each trial, and used the same cohort of fish in each predator mesocosm for each run of the experiment.

We attached 4 collapsible soft minnow traps, placed equidistantly, to the edge of each mesocosm. We ran each experiment for a 24 hr period, and replicated over 6 consecutive days, using a new cohort of trap-naïve tadpoles for each run of the experiment. Each mesocosm was lined with mosquito-netting to allow the easy removal of tadpoles after each trial.

We carried out all trials in an open field at the University of Newcastle Callaghan campus (32.886° S, 151.706° E) in NSW Australia from 10–16 May 2013. For each trial, we placed 20 *L*. *aurea* tadpoles into each of the mesocosms at 1300 h, 3 hours prior to setting traps, in order for them to acclimate to the experimental mesocosms. We added two pinches of trout pellets at this time to satiate the *G*. *holbrooki* and reduce the chance of fatal predation on tadpoles. At 1600 h we baited each trap with a yellow 13 cm glowstick (Glotek, Australia) and left them overnight. We checked traps the following morning between 0900 h and 1000 h, and recorded the number of tadpoles in each trap. We removed any tadpoles that had not been caught in the traps from the experimental mesocosms and did not use them in subsequent trials. We returned any fish that were captured in traps to the mesocosm from which they came. We exposed tadpoles to fish for less than 24 hours and used tadpoles of at least 10 mm SVL in order to reduce the risk of fatal predation [30]. No tadpoles were eaten by *G*. *holbrooki* during the experiment.

### Data analyses

Two outcome measures were used to examine capture success: capture rate (proportion of organisms caught = total number of organisms caught in the four traps / total number of organisms present in the mesocosm, for each treatment replicate) and detectability (proportion of traps occupied = number of traps in each mesocosm that were occupied/total number of traps). We used Generalized Linear Mixed Models (GLMM) to fit logistic regression models for capture rate and detectability. All statistical analyses were carried out in SAS (V9.2). Two explanatory factors (predator presence and vegetation density) were modeled as main effects and their interaction, with variability between days as a random effect to test for differences between day replicates. Significance tests were based on the likelihood ratio statistic. Odds ratios and 95% confidence intervals were calculated based upon fitted model estimates.

## Results

**Fig 2:**
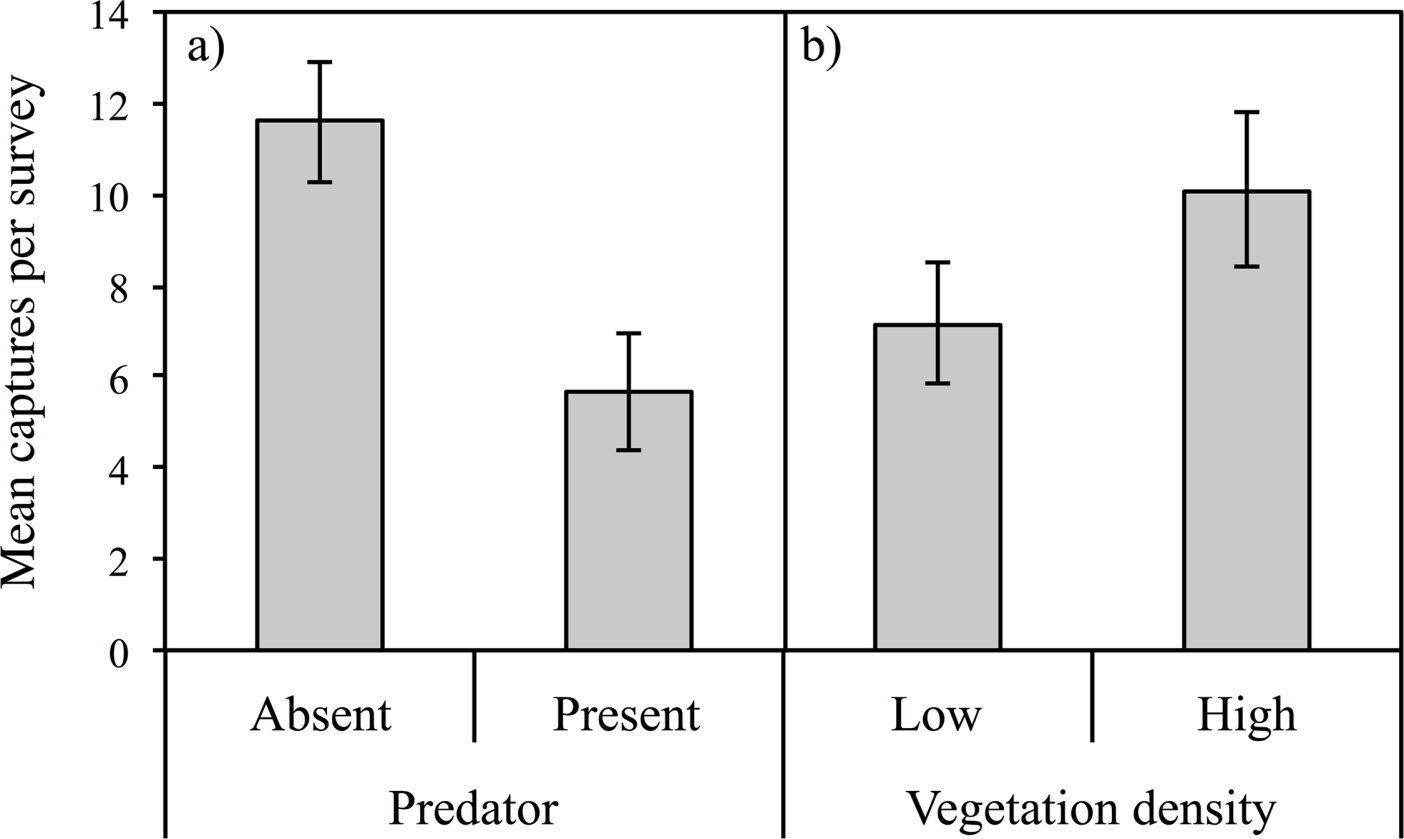
Mean number of tadpoles captured per survey for predator presence (a) and vegetation density (b), ± 1 SE. The maximum possible catch per treatment is n = 20.

The largest main effect on the tadpole capture rate was that of predator presence. In mesocosms without fish, the odds of tadpoles being captured in traps was estimated to be 4.3 times higher than that of mesocosms with fish (LR **χ** ^2^ = 45.85, d.f. = 20, *P* < 0.0001). Mean number of tadpoles captured per survey was 6 in predator present and 12 in predator absent mesocosms (Fig 2). In high density vegetation mesocosms, the odds of tadpoles being captured in traps was estimated to be 2.1 times higher than that of mesocosms with low density vegetation (LR **χ** ^2^ = 12.72, d.f. = 20, *P* < 0.01). Mean number of tadpoles captured per survey was 7 in low density vegetation and 10 in high density vegetation (Fig 2). The interaction between predator and vegetation density was not significant (*P* = 0.69).

The odds of detecting tadpoles in mesocosms without fish was estimated to be 2.9 times higher than that of mesocosms with fish present (LR **χ** ^2^ = 4.9, d.f. = 22, *P* < 0.05). Detectability of tadpoles was not significantly different for vegetation density *(P* = 0.92) or the interaction between predator and vegetation density *(P* = 0.64).

## Discussion

Our results demonstrate that two common environmental factors that vary substantially over small spatial and temporal scales significantly affect the capture success of a widespread, standardised trapping technique for tadpoles. Both capture rate and detection probability of tadpoles were affected by predator presence, although only capture rate was significantly affected by vegetation density. Tadpole behaviour has previously been shown to be affected by these two factors, however, this is the first time such behaviour has been linked to capture success for amphibian larvae and highlights the need to exercise caution when using this common survey technique in studies of amphibians. These results are similar to findings on the effects of predator presence and vegetation density on fish capture success [17, 18] and therefore suggest that such effects are likely widespread among aquatic vertebrates.

Passive trapping techniques (such as funnel trapping) are a reflection of activity and density [31]. Since density in each mesocosm was identical, the most likely explanation for these differences in capture success is behavioural plasticity, whereby the tadpoles altered their activity in response to the different environments to a point where they were less likely to encounter traps, or less inclined to enter them. Tadpoles often reduce their activity levels when exposed to predators, as this can be an effective defence against predators that locate prey through movement [19–23]. Although these past studies do not link the change in behaviour to a change in capture success, they provide a plausible explanation for the current study. If the tadpoles in the predator treatments reduced their activity levels to avoid potential predation, their chance of encountering or entering a trap would subsequently decrease. One study investigating activity levels of *L*. *aurea* tadpoles in the presence of *G*. *holbrooki* did not detect reduced levels of activity in a laboratory setting, although it is noted that the *G*. *holbrooki* were not able to interact directly with the tadpoles (leaving visual and chemical cues only to be transmitted through holes in a container) [27]. Tadpoles of some species are also known to increase refuge use when exposed to predators [24–26]. This behaviour might further explain the decreased capture success, as tadpoles may have preferentially stayed in the cover of vegetation (regardless of density) to avoid predators, instead of entering the traps, as has been reported in fish [17]. If activity is temporally and spatially constant, funnel-trap capture data can be used to estimate density differences [32], however, our results indicate that this is not the case for *L*. *aurea* tadpoles.

Tadpoles had a higher capture rate in high density vegetation regardless of predator presence. Tadpoles in the low density vegetation treatments may have recognised that they were visually more exposed to potential predators due to the sparse vegetation cover, and subsequently reduced their activity levels to lower the risk of predation [24–26], hence reducing the number of tadpoles entering traps.

An alternative explanation for our results is that behavioural plasticity was not invoked by the different environmental conditions, but rather some sort of physical block prevented location or entry to the traps. For example, *G*. *holbrooki* might have physically excluded *L*. *aurea* tadpoles from entering the traps in the predator present trials, despite an equal attempt by the tadpoles to do so compared to the predator absent trials. While this is conceivably possible, it is considered unlikely.

Regardless of the precise mechanism behind the heterogeneous capture success, it is clear that it occurred in *L*. *aurea* tadpoles, which has important implications for the use of such trapping techniques to derive metrics such as population size, or to determine presence/absence. For example, field surveys in areas with predatory fish (such as *G*. *holbrooki*) could result in a decreased capture rate of tadpoles. This bias in turn would lead to an underestimate of population size or density, or an overestimate of mortality. Furthermore, if the goal of the study was to determine the distribution of tadpoles among ponds, lowered detectability could give false absences if tadpole density is not high. Erroneous estimates of detectability are particularly problematic in endangered species research such as for *L*. *aurea*, where low density populations are frequent occurrences and false absences can be detrimental for their management [33].

One of the biggest problems created by biases in trapping success created by behavioural plasticity is the fact that the environmental cues that cause the plasticity in the first place are almost always heterogeneous across space and time. This in turn creates heterogeneous biases in capture success, as was seen in our study. Trap success may be affected by both site covariates (e.g., amount of persistent vegetation) and survey-specific covariates (e.g. water depth, water temperature, wind, time of day, observers) [34, 35]. In the present study we focused on two common factors that might influence trap success, which did not have an interactive effect. However, the natural world is rarely this simple, and interactive effects between environmental factors could be common [36]. Consequently, comparisons of abundance using funnel traps among areas varying in vegetation density and predator presence could lead to a biased estimate of spatial distribution [17].

Follow-up studies that examine the catchability of tadpoles using mark-recapture analyses would be useful to determine if bias affects all individuals equally, or if only a few change their behaviour and become trap-shy while others do not. For researchers carrying out field studies on aquatic organisms such as tadpoles, an initial trial in the field (perhaps a fenced-off area of natural ponds with a known amount of tadpoles and fish/vegetation) would be beneficial. Abundances could be measured across the field setting to adjust and account for the biases (i.e. by essentially stratifying the results). This would certainly be preferable to using absolute numbers or presence/absence without any sort of stratification. Using models to incorporate variation in detection rather than raw abundances is very important in field studies such as these, where the driver behind variation is unknown. Variation that is measured in an initial trial study can be fitted into subsequent models including the factors investigated in this study (predator presence/absence and vegetation density) as well as others (e.g., temperature, season). It may be impossible to eliminate bias from sampling if some individuals are not ever captured or detected [37], but preliminary sampling methods such as these can be employed to minimise bias.

## Conclusions

While our study has direct implications for at least one threatened species of frog (presence of *G*. *holbrooki* and vegetation density influence capture rates of *L*. *aurea* tadpoles, and should be considered accordingly), it has wide-reaching implications for trapping studies on tadpoles and other aquatic vertebrates in general. We have demonstrated that simple environmental cues can trigger behavioural plasticity in tadpoles that results in biases in trapping success. We issue a warning to researchers and surveyors that these biases may be commonplace when conducting trapping surveys such as these, and urge caution in interpreting data without consideration of such environmental factors that might be present in a given study system. Left unconsidered, these biases have the potential to lead to incorrect interpretations of data sets, and misdirection of limited resources for managing threatened and common species alike.

## Supporting Information

S1 Dataset Capture data of *Litoria aurea* tadpoles for full factorial predator presence and vegetation density mesocosm experiment. (XLSX)
